# How do nurses belonging to the LGBTIQ + community relate to patients? a qualitative study from Switzerland

**DOI:** 10.1186/s12912-025-03254-y

**Published:** 2025-05-24

**Authors:** Carole Steiger, Ursula Meidert, Andreas Gerber-Grote

**Affiliations:** 1 School of Health Sciences, Institute of Nursing, ZHAW Zurich University of Applied Sciences, Katharina-Sulzer-Platz 9, Winterthur, 8400 Switzerland; 2 School of Health Sciences, Institute of Public Health, ZHAW Zurich University of Applied Sciences, Katharina-Sulzer-Platz 9, Winterthur, 8400 Switzerland; 3 School of Health Sciences, ZHAW Zurich University of Applied Sciences, Katharina-Sulzer-Platz 9, Winterthur, 8400 Switzerland

**Keywords:** Nurses, LGBTIQ + community, Relationship building, Minority stress

## Abstract

**Background:**

The relationship between nurses and patients is particularly important, as nursing activities often require a high degree of intimacy. To promote an open, honest, reciprocal and equal relationship, nurses disclose personal information and experiences about themselves. Nurses belonging to the LGBTIQ + community, however, may fear stigmatisation or rejection from their patients if they disclose their sexual orientation or gender identity. This study aims to explore how nurses belonging to the LGBTIQ + community navigate their relationship with patients under these circumstances.

**Methods:**

A qualitative descriptive approach with semi-structured interviews was applied.

**Results:**

Interviews with eight nurses who identified themselves as members of the LGBTIQ + community, were conducted. When building relationships, nurses belonging to the LGBTIQ + community often experience a dilemma as to whether they want to come out to a patient in a care situation/encounter. When patient-carer relationships are being built, there is often a heightened focus on striking a balance between not revealing too much or too little information about themselves.

**Conclusions:**

In everyday life, nurses belonging to the LGBTIQ + community often face the dilemma of a desire for authenticity and the fear of rejection. Disclosing personal information and experiences, (to the right degree), can help build relationships.

**Supplementary Information:**

The online version contains supplementary material available at 10.1186/s12912-025-03254-y.

## Background

By definition, nursing means and entails building a trusting relationship with one’s patients. In addition to the theoretical knowledge and technical skills a nurse can obtain/learn, this is essential. In care theories such as those of Peplau [1], Travelbee [2] and Orlando [3], the interpersonal relationship between nurses and patients is attributed a very high level of importance [4]. Interpersonal relationships are at the centre of successful professional care. Successful relationship building promotes patient adherence and leads to greater satisfaction with the care provided [4, 5]. This, in turn, can have a positive influence on patient treatment outcomes [6, 7]. The consequences of nonadherence are poorer treatment outcomes and increased healthcare costs [6]. It is not only the patients who benefit from a good patient-nurse relationship; such a relationship can provide personal satisfaction, fulfilling experiences [8], and personal enrichment for nurses [9].

According to the theory of human caring [10], the nurse-patient relationship and caring itself are seen as holistic, interpersonal processes. This theory highlights the importance of an authentic, transpersonal relationship, viewing nursing as more than just a task, but as a meaningful human interaction, where empathetic and trust-based communication plays a central role. As illustrated in several caring and nursing theories, the relationship between nurses and patients is particularly important, as nursing activities often require a high degree of intimacy [4]. In addition, patients often find themselves in threatening or existential situations [4]. To promote an open, honest, reciprocal and equal relationship, nurses disclose personal information and experiences about themselves [11]. The person-centred approach, according to Rogers [12, 13], is a widely used model in nursing for successfully building relationships with patients [14, 15]. Rogers emphasises the importance of empathy, congruence, and the unconditional appreciation of the nurse toward the patient [12, 13]. The aim of the person-centred approach is to create a supportive environment for the patient in which he or she can develop and self-actualise [12, 13].

According to Meyer’s minority stress model, people belonging to the LGBTIQ + community must always expect prejudice, discrimination and rejection [16–18]. Meyer’s minority stress model [16–18] is a fundamentally theoretical model, which assumes that society is heteronormative. This means that heterosexual orientation and cisgender identity are seen as “normal” and “right” and that other forms of sexuality and gender identity are considered “deviant” and “wrong” [16–18]. This can lead to various forms of stress [16–18]. Distal stressors can include discrimination, stigmatisation, violence and bullying on the basis of gender identity or sexual orientation. Proximal stressors include self-stigmatisation, hiding one’s sexual orientation and gender identity or the feeling of not being able to live authentically. Management of emotions to meet the emotional demands of a job is known as emotional labour [19]. Nurses in particular do not merely provide professional care, but are meant to foster positive emotions such as optimism. They are also meant to provide reassurance, even though inwardly they may feel differently.

These stressors may result, as Meyer [16–18] described, in (various) health problems. Resulting health problems may include chronic illnesses, an immune deficiency, psychological problems such as anxiety, depression or addiction, as well as isolation and problems establishing and maintaining relationships. Studies have shown, that negative reactions from work colleagues and superiors can lead to stress, depression, internalised stigmatisation, loss of confidence, anxiety or a negative attitude toward work [20]. It can be assumed that the same would be the case if negative reactions were to come from clients or patients, as they are part of the work environment.

The term “LGBTIQ+” unites lesbian, gay, bisexual, trans, intersex, non-binary and queer people [21]. This refers to various dimensions of a person, including sexual orientation, gender identity or gender characteristics [21]. In Switzerland, approximately 13% of the population identifies as part of the LGBTIQ + community [22]. Worldwide, data confirm that about 11% of the population identifies as part of the LGBTIQ + community; however, this number varies by generation, region, and culture [22]. Gen Z individuals are approximately twice as likely as millennials and four times as likely as Gen X and Baby Boomers are to identify as bisexual, pansexual, omnisexual, or asexual [22].

Although a recent survey has shown that people belonging to the LGBTIQ + community are discriminated less in Switzerland than in any other participating country [22], the situation in Switzerland shows that people belonging to the LGBTIQ + community continue to face prejudice, discrimination and structural inequality [23]. Compared with the rest of the Swiss population, people belonging to the LGBTIQ + community have less pronounced social and psychological protective factors [24]. The reason for this is seen in internalised homonegativity or trans-negativity [24]. According to Krüger et al. [24], these internalized negative feelings increase with experiences of discrimination. Although major political and social changes are underway, those belonging to the LGBTIQ + community do not feel accepted everywhere in Switzerland [23]. Experiences of discrimination, harassment and violence, of isolation and exclusion are prevalent in the LGBTIQ + community all over Europe [25].

This study focuses on congruence, as it contradicts the minority stress model described by Meyer [16–18], which shows that people belonging to the LGBTIQ + community can feel that, in nursing care situations, they are not allowed to be themselves or disclose an integral part of their identity. The aspect of their sexual orientation or gender identity can arise in many situations, in which these aspects are either explicitly addressed or implicitly brought up by a patients’ comments, questions, or behaviour. This may include, for example, stereotypical assumptions, curious inquiries, or rejective attitudes / reactions.

In this study, we aim to shed light on the following question: How do nurses belonging to the LGBTIQ + community experience and organise/foster a professional relationship-building with patients, when their sexual orientation or gender identity come up in encounters of care and they may consequently face rejection or stigmatisation from patients.

## Methods

A descriptive explorative study design was used. Semi-structured interviews were conducted to allow participants to provide in-depth information about their feelings, attitudes, and ways of navigating patient relationships [26]. Interview guidelines are provided as a supplementary file. No definitive assumptions were made in answering the research question; rather, an open-ended, exploratory approach was adopted. It was considered plausible that identifying as part of the LGBTIQ + community might influence nurse-patient interactions and potentially act as an additional stressor. Furthermore, it was assumed that participants would employ diverse strategies in building therapeutic relationships, including those related to managing their gender identity and/or sexual orientation. No assumptions were made regarding the extent or nature of the participants’ experiences.

### Settings and participants

Potential participants were people who feel that they belong to the LGBTIQ + community and who work as nurses. Inclusion criteria were self-identification as a member of the LGBTQ + community, current employment of at least two working days per week, minimum of one year of clinical practice experience, sufficient German language skills for nuanced expression and legal age (≥ 18 years). Exclusion criteria were identification as a heterosexual cisgender woman or man, current work engagement below two working days per week or unemployment, less than one year of clinical practice experience, insufficient German language skills or being underage (< 18 years).

Access to the sample was gained by contacting numerous hospitals, nursing homes, rehabilitation clinics, home-care services, and psychiatric centres in the Northwest of Switzerland. Organisations were asked to distribute information about the study to their staff. The study information was shared with staff via the respective organizations’ internal communication systems. It included project description, project aim, inclusion criteria, information on data collection, ethical information, information on data protection, institutional framework of the study and information on the authors.

Eight nurses responded to the information letter. At the time of the interview, participants were working as nurses in homecare, long-term care or a psychiatric clinic (see Table 1). All participants had at least one qualification as a healthcare professional and had been working in nursing for at least seven years. Participants were between the ages of 20–57 years. They identified themselves as cis women, cis men, trans men or non-binary.

**Table 1:** Study participants

	Identification in the LGBTIQ + spectrum	Work setting
1	Homosexuality	Psychiatry
2	Trans identity	Outpatient care
3	Homosexuality	Long-term care
4	Homosexuality	Long-term care
5	Panromanticism/asexuality	Psychiatry
6	Homosexuality, also sees itself in non-binary	Psychiatry
7	Homosexuality	Outpatient care
8	Homosexuality	Long-term care

### Ethical considerations

The study was reviewed and approved by the ZHAW Ethics Committee (EA-ZHAW-2023-STA-016-G). As part of the consent process, participants were informed that their participation was entirely voluntary and that they could withdraw their consent at any time without providing a reason. They were reassured - both in writing and orally - that all data would be treated confidentially and that results would be presented in a way that ensured their anonymity. Signed consent forms were obtained before participants were included in the study to protect participants’ identities. All identifying information was removed from the transcripts, and the data were securely stored in a password-protected computer. The audio files and the data key were irrevocably destroyed after completion of the analysis.

### Data collection

For the data collection, CS conducted eight semi-structured single interviews in October and November 2023. No prior relationship existed between the nurses and researchers. The interviews lasted between 35 and 90 min. They took place at a location chosen by the participants. The interviews were audio-recorded. Field notes were taken during data collection to record spontaneous thoughts and ideas, which were also integrated into the data analysis [27].

### Data analysis

The interviews were then transcribed using the transcription rules of Dresing and Pehl [28]. The data was analysed using the MAXQDA^®^ software [29]. On the basis of content analysis according to Mayring [30], specifically summarizing content analysis, several categories were formed both deductively and inductively. To identify central themes and patterns, text passages were then systematically assigned to the relevant categories and interpreted in terms of content.

### Rigor strategies

To ensure the rigor and high quality of this study, several quality criteria were considered. Objectivity was maintained through detailed procedural documentation following the guidelines set by Mayring [30], which ensure transparency and replicability of the research process. Furthermore, participants were given the freedom to choose the location of the interview, creating a familiar and comfortable environment that facilitated an open and unrestricted expression of their thoughts. The study was conducted as part of a master’s thesis. Therefore, coding was only conducted by CS. However, reflections were regularly held with fellow students, AGG and UM. These reflections included aspects like the method of conducting interviews, as well as an analysis of the influence of the interviewer’s own gender identification, sexual orientation and attitude on the interview/interviewee.

## Results

In this section, we address major issues related to the experience of building relationships with patients, navigating communication and disclosing or not disclosing LGBTIQ + identity to patients. In the analysis, two main categories and several subcategories emerged (see Table 2). We first established the categories and then moved on to support our results with quotes from our samples.

**Table 2:** Categories made

Main categories	Subcategories
The experience of relationship building with patients	Successful coming out Unsuccessful coming out Presumed patient attitudes Self-stigmatization
External factors influencing relationship building	Society, politics and preconceptions The team

### The experience of relationship building with patients

The participating nurses consciously thought about what personal information they would and would not share with patients in order to build or maintain a professional relationship, keep a professional distance and protect themselves. What information to be disclosed is personally determined by the participants and varies by person. This includes topics such as point of residence, their hobbies, but also more specifically their relationship status and their sexual and romantic orientation. Participants ensure that they provide the same information to all patients, as patients may share this information with each other, especially in an inpatient setting.

In order to build a relationship, some information may also be disclosed to just one patient rather than another, thereby uncovering a commonality and creating trust.

Targeted with the emphasis on targeted. Not in principle. Basically, I’m not the subject; my patient is. […] and if I realise that the (patient) has a bit of a problem trusting me, then I might reveal something about myself. (Nurse 6)

Some participants assume that they are more concerned about the issue of disclosing personal information to patients than their cis-hetero colleagues. However, no one from our sample discussed this issue with their colleagues or with their team.

Participating nurses are aware of the patients’ desire to establish or deepen their relationships with them. A way of doing so is asking questions about their private life. Patients want to get an idea of who is caring for them. This type of relationship building is important because, as a nurse, you know a lot about the patient and some very personal information is exchanged. In order to build trust, it is therefore understandable that patients also want some information about the nurses caring for them.

So patients always ask. Because they want to build a relationship with you […]. So you come in every day, you always see the same people. They get to know you after a while. And they’re also curious. […] They also want to get a picture of someone. And then, of course, they ask where you live, how old you are, what kind of education you have, are you married, are you not married, do you have children. That is also interesting for them and that is also important. I think it is important to have a relationship and to get an idea of who you are. That is part of the person. And that is why they ask that. (Nurse 3)

Patients probably do not realise that, with seemingly harmless questions about private life, they can cause a dilemma for nurses from/belonging to the LGBTIQ + community. Patients often assume that nurses belonging to the LGBTIQ + community are cis men or cis women and heterosexual. Almost all study participants had already experienced situations or phases in their lives in which they answered questions evasively or changed the gender of their partner in the story they told. Participants, however, stated several times that they did not want to lie.

And I was lucky there, I found quite a good relationship with her (the patient). […] And then she also asked me if I had a boyfriend. And I had a girlfriend at that moment. And then I just said “No, I do not have a boyfriend”. That is the truth. And she then said, “Oh no, that is a shame, but you’re such a great girl. You will find the right one”. I left it at that. But I did not lie at that moment either. […] I do not have a boyfriend. In order to maintain the relationship, I simply answered that way. (Nurse 5)

Most of the questions focused on relationship status and family life. One exception is the participant who is a trans person. In this case, some of the patients’ questions focused on the topic of gender reassignment. Some of these questions were very personal.

### Successfully coming out/successful IN coming out

When coming out, participants say that they are anxious, tense or amused as they wait for the other person’s reaction. The initial concern/suspicion that a negative reaction would follow, often does not materialise.

Did not materialise (suspicion of a negative reaction) at all. Many simply take note of it and say “yes”. Or just where it was around the vote (marriage for same-sex couples in Switzerland, 2021). “Yes, we’re allowed to do that now”. […] But it is not that I need to talk more about it. Or I do not think any more than I would if I had a man at home now. (Nurse 7)

Reactions, however, vary greatly. For the most part, they are positive or neutral. This may be because openness and/or trust is appreciated by patients, they may also simply feel or regard this information as normal.

### Unsuccessfully coming out/unsuccessful IN coming out

In general, participants reported few conflict situations. Two participants also explicitly stated that they had not yet experienced a conflict with a patient due to their sexual or romantic orientation or gender identity.

And I think they (certain patients) would stigmatise me as “you’re a lesbian”. And that would be the problem. And not the way I work. It is just that. And I think that is just such a fine line. Or my inner knowledge that it could happen. Or that it will happen. Or maybe it has already happened, and I just have not realised it. (Nurse 1)

Verbal insults, a lack of understanding and the breakdown of relationships and support were cited as conflicts. Clichés and stigmatisation, not only from patients but also from team members, were also experienced by some participants. An example of this is the belief that in same-sex couples, one person must take on the role of the woman and the other person the role of the man. Two participants reported that patients rejected care and support after a coming out by the nurse, even verbal abuse and insults were experienced.

I have already been not allowed to look after one patient. Because he said, he did not want to work with a lesbian or even with gays. (Nurse 1)

Disclosure could trigger a discussion or negative reactions, which they want to avoid. Participants do not want to justify themselves or discuss their own sexual or romantic orientation or gender identity with patients.

And I do not have to make my life any harder than it is. And I was rather afraid that there would be negative reactions from the patients. And that I would not enjoy my work anymore. That I would no longer enjoy coming to the ward if I had to expect negative reactions from patients all the time. (Nurse 5)

In addition to protecting themselves from negative reactions, it is also important for participants to protect patient. They do not want to upset the patient’s beliefs and values. Another fear is that, because of rejection, a therapeutic relationship will no longer be possible. Consequently, certain participants refrained from disclosing their sexual orientation or gender identity in order to preserve an existing relationship.

However, the most frequently cited reason why participants do not come out in care situations, or generally in everyday working life, is that it does not seem necessary in the given situation or for the relationship at that moment.

[…] so I just did not find it necessary to come out there. I’m in a professional relationship there. I care for the patient, and I actually took the same position. So, if I had been heterosexual, I would not have stood in front of the patients and talked about my boyfriend or my husband. (Nurse 4)

### Presumed patient attitudes

Certain participants, depending on the age and presumed attitude of the patient, differentiated regarding a coming out.

Before, that is actually before marriage for all was accepted in Switzerland, I did not say it to everyone like that. Especially with the very old or where I knew they were very conservative. I did not say much of anything when they were asked, “Where does your husband work?“. Then, I said, “He also works in the healthcare sector” and did not go into it any further. (Nurse 7)

Older people grew up with a more negative image of homosexuality. In previous generations, the topic of trans identity was rarely discussed. Participants therefore assumed that coming out could lead to greater problems. They also expressed a great deal of understanding for the fact that patients react less positively to coming out because of what they have been taught about homosexuality and trans identification. Several participants reported that they were also met with a surprisingly high level of tolerance and openness from older patients. The following statement by a trans- person makes this point:.

[…] last time a client said it truly suits me, being a man, and that is nice. So to still meet with so much acceptance. Although you just do not expect it from this generation. (Nurse 2)

Experience shows that younger patients, being more in touch with LGBTIQ + issues, tend to see variations in sexual orientation and gender identity as normal. Naturally, patients who consider themselves part of the LGBTIQ + community are not treated differently by the participants. Participants, however, assumed/believed that these patients have a greater understanding for them regarding certain areas of life or could even support younger patients in the discovery process. Two participants also expressed a certain discomfort, as they found it more difficult to maintain a professional distance when caring for patients belonging to the LGBTIQ + community.

I still have to make sure that it remains professional. And I’m truly scared when I say “hey, I can understand this one hundred per cent”. I do not know. That it will then degenerate into friendship and no longer be professional. It is extremely difficult to find the right closeness there. (Nurse 5)

According to the participants, illness-related behavioural changes in patients also have an influence on the relationship. Mental illnesses and cognitive impairments can lead to reduced emotional regulation or manipulative behaviour, among other things. For this reason, participants tend to be more cautious with respect to conveying personal information, such as their own sexual and romantic orientation and gender identity.

### Self-stigmatisation

Two participants described feeling guilty, which they did not consider rational (when caring for patients) during intimate care activities. One participant disclosed her sexual orientation to the patients, while the other did not. The participant who disclosed her sexual orientation was worried that she could be accused of sexual molestation. or that patients would feel uncomfortable because she had openly communicated her sexual orientation.

That when I come in (to the patient’s room), my fear is that you (the patient) will feel uncomfortable. Maybe that will not happen. But I want to do it right for everyone. Because I am different. (Nurse 1)

The participant who did not disclose her sexual orientation wondered whether she was obliged to do so. She wondered what the patients would think if they found out about her romantic interest in women afterwards.

For the participants, the desire not to lie and to live their sexual and romantic orientation openly speaks in favour of coming out.

And I realise it is not right for me not to come out, not to say how I live. (Nurse 6)

External factors influencing relationship building.

### Society, politics and preconceptions

The vote on same-sex marriage in Switzerland in 2021 has led to certain participants communicating their relationship status and sexual orientation more openly, including in contact with patients. They describe a more open communication brought about by the clear acceptance of the voting population and the support of society. A contributing factor was also that the vote brought a large part of the Swiss population into contact with the topic.

In this spirit, participants wish to achieve a greater visibility of the topic of different sexual orientations and gender identities and that it is normal and nothing unusual. It was also mentioned that, when working with younger patients in psychiatry wards, nurses want to support patients in their discovery phase. If a nurse is open regarding her sexual orientation, the inhibition threshold to address this topic can be lower. The desire for transparency was also mentioned. In the case that patients know about the LGBTIQ + affiliation of the carer from the outset, they can decide whether or not they want to establish a relationship. This will prevent the relationship being broken off at a later point in time. In addition to the aspect of transparency, it can be said that open communication prevents speculation on the part of the patient.

And I think it is precisely to prevent this (relationship breakdown) that I want it to be transparent from the start. I know it can backfire. […] Then, you can decide whether you want to work with me or not. Do you want to enter into a relationship with me or not. If it is such a big problem for you, you know it from the start. Yes, then stay away from me. (Nurse 1)

Overall, participants stated that the LGBTIQ + community is very diverse and that many cis-hetero nurses as well as the general population are not aware of the challenges, they face in navigating their way through society. One aspect is that everyone has an opinion about the LGBTIQ + community, including patients. This is why they often face preconceptions and stereotypes about being a member of this community.

I do not think many people realise this, but we (people belonging to the LGBTIQ + community) are subject to constant judgment. Yes, people either hate us or like us. […] People have an opinion about it. You always have to deal with the fact that that can happen. (Nurse 3)

### The team

All participants expressed the importance of support from their nursing team. They would like the team to have an open, tolerant attitude in which their sexual and romantic orientation and gender identity are perceived as normal or even as a resource. This open attitude provides participants with the security of knowing that they can count on the support of their team in the event of a conflict between themselves and the patients. Normalisation, therefore, can make it easier to build relationships with patients.

It is also much more difficult to build relationships with my patients if it is made problematic by others. And if it is not made problematic by my team, it is also easier for me to establish a relationship with patients. Or to get there at all. I think that is why they (the team) are so relevant. Or very relevant for me. (Nurse 1)

Some participants reported curiosity, ignorance and uncertainty among their colleagues when dealing with LGBTIQ + people.

## Discussion

In our qualitative study, we investigated how nurses belonging to the LGBTIQ + community experience and shape relationship building with patients and navigate between congruence, authenticity and the fear of rejection due to their gender identity or sexual orientation. The results show that nurses belonging to the LGBTIQ + community are faced with a dilemma when building relationships and deciding whether they want to come out. These experiences are quite emotional for the nurses being interviewed in our study. Considerations and reflections at the level of the emotional relationship with the patients pervade many of the aspects expressed in the interviews. Nurses reported feeling that they were in a dilemma between authenticity and feared rejection. The desire for authenticity, as delineated in Rogers’ person-centred approach [31], stipulates the motivation of not wanting to hide and wanting to disclose sexual orientation or gender identity to patients. Several participants indicated that they do not wish to deceive patients and expressed a preference for not providing evasive responses. Participants also wanted to create visibility for the topic and support patients belonging to the LGBTIQ + community.

Disclosing personal information can help in building trust. Nurses use this as a technique for relationship building. According to Unhjem et al. [11], nurses disclose personal information and experiences about themselves to promote an open, honest, close, reciprocal as well as equal relationship. Patients often ask personal questions, which shows their desire to build a relationship by wanting to know who this other person is. This desire can be explained by the fact that nursing activities often require a high degree of intimacy, and patients often find themselves in threatening or existential situations [4]. It can also be seen as a way to establish reciprocity in an unequal relationship, in which the nurse knows much more about the patient than the patient does about the nurse. Unhjem et al. categorised the self-reports of nurses [11] into immediate family, interests and activities, life experiences and identity.

Participants carefully considered which information they wanted to disclose to the patients. In terms of immediate family, they regard disclosing their sexual and romantic orientation or gender identity and lifestyle as a challenge, as this may be seen as a diversion from a heteronormative one. This process is often accompanied by emotions. Nurses perceive it as a potential for conflict. Unhjem et al. [11] described the challenges faced by nurses when patients ask questions about their private lives. Only a few nurses refuse to answer, but they endeavour to strike a balance between not providing too much and not too little information [11]. This method has been described by Goffman [32] as “techniques of information control”. According to Goffman [32], these techniques to protect identities when they deviate from approved standards, mainly through concealment, are sometimes elaborate.

Study participants also expressed an understanding for being asked personal questions, as most patients tell a lot about themselves without having any information about the nurse they confide in. Nurses may wish to protect their privacy and sometimes do so by limiting themselves to details when answering patients’ questions [11]. Study participants also pointed out that they try to answer questions evasively. By doing so, they strike a balance between answering the question without lying and not having to come out in this situation. Particularly when the topic of their own partnership or sexual or romantic orientation came up, participants reported that they avoided these questions.

The fear of rejection is a direct result of various negative reactions by patients and negative comments regarding the LGBTIQ + community. Participants reported negative reactions such as insults and relationship breakdown. In order to prevent/forestall these negative reactions, they decided against communicating their sexual and romantic orientation.

The feeling of having to deny one’s sexual identity was recognised by one participant in that she behaved overcautiously when in intimate care situations. She did this out of fear that patients might feel distressed because of her disclosed homosexuality. The participant described how she knows that, in a moment like this, she is looking at the situation professionally and, for fear of reproach, reduces herself to her sexuality.

A motive for not coming out was not wanting to shake the other person’s world views and beliefs, as well as the wish to protect patients. Under the pretext of protection, nurses avoid being confronted with the dilemma of coming out and possible negative consequences. Avoidance can therefore also be interpreted as self-protection. Another possible interpretation could be that nurses feel responsible for the fact that coming out could lead to a conflict situation. They see it as their responsibility not to disclose any information that is potentially unpleasant for the patient. This relates back to the emotional labour that nurses perform in professional relationships (Hochschild 1983).

As emphasised by Hässler & Eisner [23], participants notice the prevailing social shift/change in Swiss society. At the same time, they experience the stress described in the minority stress model [16–18]. External stressors, including the deterioration of the relationship with a patient, as well as prejudice and stigmatization, alongside internal stressors such as self-stigmatization, were identified. Many patients also carry prejudice and stigmatization within them, which the participants recognise from their statements. In patient contact, heteronormativity is recognized by participants in the fact that patients often assume that they are cis men or cis women and heterosexual. Within the team, certain participants noticed their colleagues’ curiosity, lack of knowledge and uncertainty about how to deal with LGBTIQ + people.

### Strengths and limitations

To our knowledge, this is the first study to investigate how nurses, in a relationship with their patients, deal with the dilemma of transparency and personal identification with their LGBTIQ + identity.

Our study has several limitations. The study was conducted in only one part of German-speaking Switzerland. Experiences may differ in other parts of the country (more rural or urban) or in other countries. Furthermore, only one person who did not come out when in contact with patients took part. It can be assumed that the hurdle of taking part in a study is much higher for people who do not openly communicate their sexual orientation or gender identity. Several efforts were made to minimize barriers of participation. Anonymity of participants, for example, was clearly emphasised in the information material distributed to potential participants. It is also possible that more people are opening up to their sexual orientation or gender identity due to social change.

No person currently working in a hospital took part in the study. Several participants have worked in a hospital during the course of their career and were able to contribute to this perspective. It’s therefore possible that experiences of nursing staff working in a hospital was not fully depicted.

Recruitment material was sent via the institutions. In institutions where superiors did not want to support the study, study information was not forwarded to the nursing staff. It is possible that institutions which considered the topic to be of little importance did not forward the study.

The attitudes of an institution can have a negative influence on the well-being of the nurses, therefore also on their ability to build trusting/meaningful/caring relationships with patients. Other paths, without the involvement of an institution, could have been used to reach study participants. The recruitment of participants via healthcare institutions was chosen to ensure access to a sufficient number of nurses. Without this institutional support, it would likely have been challenging to reach an adequate sample size. However, the potential influence of this recruitment strategy on the study findings remains unclear.

In one interview, a technical problem resulted in a loss of data. This was recognised immediately after the interview and as much of the interview content as possible was recorded as field notes. Nevertheless, it is likely that there was a loss of information which could have had an impact on the results.

## Conclusions

When building relationships, nurses belonging to the LGBTIQ + community often experience a dilemma regarding coming out to a patient. This dilemma is usually triggered by questions from patients about the nurse’s private life. Various parts of the private life of nurses, such as family and leisure time activities, are impacted by their gender identity or sexual orientation. Patients’ questions serve to build relationships and trust, which is why nurses do not want to desist them. Nevertheless, nurses belonging to the LGBTIQ + community must also expect rejection if they come out. Strategies employed by nurses in such situations vary. Some consistently disclose their sexual orientation or gender identity and subsequently observe the patients’ reactions while others may adopt a more evasive approach, such as using gender-neutral language or aligning the gender of their partner with heteronormative expectations, in order to avoid potential discomfort or conflict. When building a relationship, there is often an increased focus on not revealing too much or too little information about themselves. Coming out, however, can also be a strategy to building a trusting relationship with patients.

### Study implications

For the nursing practice, study results show that it can be useful for nursing teams to develop an accepting and supportive attitude toward nurses belonging to the LGBTIQ + community. This attitude helps nurses cope in situations of conflict with patients. A supportive attitude/climate could be promoted by sensitising care teams through workshops and training, and an inclusive policy by employers. Healthcare institutions should establish clear guidelines that protect sexual and gender diversity in the workplace and should ensure that discriminatory behaviour by patients or staff is not tolerated. At the policy level, national frameworks could support inclusion by integrating LGBTIQ + sensitivity into nursing education and professional standards.

The phenomenon of a perceived need to protect the patient could be investigated further, ideally using a phenomenological approach to gain deeper insight into its underlying meaning and implications.

## Electronic supplementary material

Below is the link to the electronic supplementary material.

Supplementary Material 1

Supplementary Material 2

## Data Availability

Data files in German are available upon request to the corresponding author.

## Abbreviations

Nurses: Registered nurses
