# Supplementary material for: How do nurses belonging to the LGBTIQ + community relate to patients? a qualitative study from Switzerland
Source: BMC Nurs. 2025 May 24;24:586. doi: 10.1186/s12912-025-03254-y (PMC12103751; doi:10.1186/s12912-025-03254-y)
Supplement: Supplementary file 1 — Supplementary Material 1 [file 12912_2025_3254_MOESM1_ESM.docx]

# Interviewleitfaden

### Vorbereitung Interview

- Danksagung für die Teilnahme
- Ablauf des Interviews und Zeitspanne aufzeigen
- Einleitung (Erklären, was ich erforsche und was von der Person erwartet wird)
- Erläutern, dass die Person Fragen nicht beantworten muss, wenn sie nicht möchte, das Interview jederzeit pausieren oder abbrechen darf, wenn die emotionale Belastung zu hoch wird.
- Unterzeichnen Einverständniserklärung
- Per Du oder per Sie?
- Wichtigste Informationen wiederholen (Widerrufsrecht, Anonymität, Aufnahme)
- Fragen klären

Einstiegsfrage

- Als du von dieser Studie gelesen oder gehört haben, was ging dir durch den Kopf?

Fragen für den Vertrauensaufbau im Interview

- Die folgenden Fragen sollen den Vertrauensaufbau stärken und werden je nach dem, wie der Gesprächsfluss ist gefragt oder direkt zum Hauptteil übergesprungen.
- Was gefällt dir besonders am Kontakt mit den Patient:innen?
- Was ist deine Strategie um neue berufliche Beziehungen mit Patient:innen aufzubauen?

Hauptteil

Schauen wie sich das Gespräch nach den Fragen vom Beginn entwickelt. Wenn das Gespräch zum Stillstand kommt, kann auf folgende Fragen vertiefter eingegangen werden:

- Wie würdest du deinen Beziehungsaufbau zu Patient: innen beschreiben?
- Wie ist dein Umgang mit deiner sexuellen Orientierung / Geschlechtsidentität im Kontakt mit Patient: innen?
- Hast du in Beziehungen zu Patient:innen das Gefühl du selber sein zu können?
- Wie spürst du dass du du selbst sein kannst im Kontakt mit Patient:innen?
- Was beeinflusst deiner Ansicht nach den Beziehungsaufbau?
- Wie schützt du dich vor verletzenden Situationen im Kontakt mit Patient: innen?
- Wie kann dich das Team / der Arbeitgeber unterstützen?

Konkretisierungsfragen / Vertiefungsfragen:

- Wie erging es dir in dieser Situation?
- Wie hast du diese Situation wahrgenommen?
- Was hat das in dir ausgelöst?
- Wie hast du darauf reagiert?
- Kannst du das für mich noch einmal wiederholen?
- Kannst du das noch konkreter / detaillierter erzählen?

Ende

- Demografische Daten falls noch nicht erwähnt (Geschlechtsidentität, sexuelle Orientierung, Arbeitssetting, Arbeitserfahrung, Alter)
- Gibt es noch etwas zu dem Thema, was du noch gerne sagen würdest und noch nicht dazugekommen bist?
- Gibt es einen wichtigen Aspekt, welcher noch nicht zur Sprache kam?

Abschluss

- Noch einmal bedanken für die Teilnahme
- Erklären was mit den Daten passiert
- Fragen, ob die Person eine Kopie der Masterarbeit möchten (Falls ja: E-Mail-Adresse einholen)
