# Supplementary material for: How do nurses belonging to the LGBTIQ + community relate to patients? a qualitative study from Switzerland
Source: BMC Nurs. 2025 May 24;24:586. doi: 10.1186/s12912-025-03254-y (PMC12103751; doi:10.1186/s12912-025-03254-y)
Supplement: Supplementary file 2 — Supplementary Material 2 [file 12912_2025_3254_MOESM2_ESM.docx]

# Interview guide

Opening and Welcome

- Thank the participant for their participation
- Provide an outline of the interview and the expected duration
- Introduce the study (explain what the aim of the study and what is expected of the interviewee)
- Emphasise that participants are under no obligation to answer any question they are uncomfortable with, and that they may pause or terminate the interview at any point if they feel emotionally overwhelmed
- Signing of the consent form
- Inquire about the preferred way to address the interviewee
- Repeat key information: right to withdraw, anonymity, audio recording)
- Clarify any questions the interviewee may have

Introductory question

- What came to your mind when you first read or heard about this study?

Rapport-Building questions

The following questions are intended to build trust and are asked depending on the flow of the conversation or skipped to continue directly with the main part.

- What do you particularly like about your contact with patients?
- What is your approach to establish new professional relationships with patients?

Main part

After the opening questions the interviewer continually checks the flow of the conversation. The following questions may be used to maintain the flow of the conversation, to deepen the dialogue or to prevent a standstill:

- How would you describe how you build relationships with patients?
- How do you navigate your sexual orientation/gender identity in your interactions with patients?
- Do you feel that you can be your authentic self in your relationships with patients?
- What makes you realize that you can be yourself in patient interactions?
- What do you think influences relationship-building in your role?
- Which strategies do you use to protect yourself from psychologically harmful situations in contact with patients?
- In what ways can your team or employer support you to handle such situations.

Questions to become more specific / Follow-up questions

- How did you feel in that situation?
- How did you perceive that situation?
- What did this event/ experience/ situation trigger in you?
- How did you respond?
- Could you repeat that for me again? Did I understand that right…? (in order to clarify if something was not clear)
- Could you explain that in more detail? (situation, emotion, etc.)

Towards the end of an interview

- Demographic data, if not already mentioned (gender identity, sexual orientation, work setting, work experience, age)
- Is there anything else you would like to share on topic that you haven't covered yet?
- Is there any important aspect that has not yet been addressed?

Closing

- Thank the interviewee for their participation
- Explain what will happen with the data collected
- Ask whether the participant would like to receive a copy of the completed master's thesis
